# Management of children with hemophilia A on emicizumab who need surgery

**DOI:** 10.3389/fped.2023.1155853

**Published:** 2023-04-04

**Authors:** Mark Belletrutti, Mihir Bhatt, Nasrin Samji

**Affiliations:** ^1^ BC Children’s Hospital, Oncology/Hematology/BMT, University of British Columbia, Vancouver, BC, Canada; ^2^ McMaster Children’s Hospital, Hematology/Oncology, McMaster University, Hamilton, ON, Canada

**Keywords:** hemophilia A, emicizumab, surgery, bleeding, pediatric

## Abstract

The introduction of emicizumab into the treatment regime of persons with hemophilia A has dramatically reduced frequency of bleeding in patients with and without inhibitors. However, in children with Hemophilia A (CwHA) who require surgical or other invasive procedures, additional treatment with factor replacement or other hemostatic agents may still be needed to prevent intraoperative or postoperative bleeding. This review will look at the reported outcomes in CwHA on emicizumab who have had surgery and propose recommendations for the best perioperative management of major and minor procedures.

## Introduction

Hemophilia A is a congenital deficiency of clotting factor VIII (FVIII) due to the presence of mutations on the FVIII gene. It is an X-linked disorder primarily affecting males and results in a lifelong bleeding tendency. Frequent, seemingly unprovoked bleeding into joints and muscles as well as potentially life-threatening bleeding into vital areas (such as the central nervous system) can lead to significant long-term complications (1). The mainstay of treatment is FVIII replacement using purified factor concentrates. In many areas of the world, prophylactic FVIII replacement to prevent bleeding is the standard of care, especially in children (2, 3). While effective at preventing most bleeding episodes, FVIII prophylaxis comes with considerable challenges in the pediatric population, including development of inhibitors as well as need for venous access, which often requires surgically implanted catheters to support the frequency of regular prophylactic intravenous infusions (4).

In up to 30% of previously untreated children, the development of FVIII neutralizing autoantibodies (inhibitors) renders FVIII replacement ineffective (5–7) and requires eradication of the inhibitor through immune tolerance induction treatment (8). Until eradication, episodic bleeding is treated with alternative agents that bypass the FVIII step in coagulation (BPA)(activated prothrombin complex concentrates [aPCCs], recombinant factor VIIa [rFVIIa]) (9–11) which, unfortunately, have suboptimal and unpredictable efficacy. This leads to increased bleeding episodes, increased morbidity and mortality, and decreased quality of life (12, 13).

Emicizumab (Hemlibra®, Roche, USA) is a bispecific humanized monoclonal antibody engineered to bring activated factor IX and factor X together allowing activation of factor X thus mimicking the action of missing FVIII in persons with hemophilia A (14). Restoration of FVIII function with emicizumab results in activity at 10%–20% equivalency of endogenous FVIII (15, 16) and converts an individual from a severe bleeding phenotype to a mild phenotype. Emicizumab has shown efficacy in bleed prevention in both adolescents/adults and children with Hemophilia A with and without inhibitors (17–20) and has the added benefit of subcutaneous administration, removing the need for venous access. This has led to widespread adoption of emicizumab as prophylaxis therapy in many countries (21).

Many children with Hemophilia A require surgical procedures throughout their lifetime, often because of their disease. In addition, routine procedures such as dental extraction or tonsillectomy can also lead to excessive bleeding if not managed appropriately. Coordination between the Hemophilia Treatment Center and surgical teams is essential to ensure proper treatment can be given prior to, during, and after surgery to minimize bleeding complications. This remains the standard approach in the emicizumab era as additional factor replacement or treatment with other hemostatic agents may still be needed. To date, no standard guidelines exist to guide clinicians on what the best practice is for preparing children with Hemophilia A on emicizumab for surgery. However, data on surgical outcome from the seminal clinical trials and from published real-world experience can guide clinicians on how to manage their patients. This review will appraise the data from these publications and present recommendations for laboratory testing, treatment for major and minor procedures. Additional unanswered questions will also be discussed.

## Methods

A PubMed search using the terms “emicizumab AND surgery” was conducted. Relevant journal articles were selected, and reference lists reviewed for additional publications. All articles containing pediatric patients 0–18 years of age where data could be easily extracted were included. Articles with only adult patients or where pediatric data could not be easily separated or interpreted were excluded.

Major and minor surgical procedures were defined based on Santogostino et al. (22) Minor surgery was defined as an invasive procedure involving manipulation of only skin, mucous membranes, or superficial connective tissue. Major surgery was defined as an invasive procedure which included one or more of the following: entering a body cavity, crossing a mesenchymal barrier, opening a fascial plane, removing an organ, or operatively altering the normal anatomy.

Post-operative bleeding definitions were based on the HAVEN studies (23) and were defined as bleeding that occurred after surgery and felt to be “due to the surgery/procedure.” A treated bleed was defined as a bleed that was directly followed by the administration of a FVIII concentrate or rFVIIa, irrespective of the time between the treatment and preceding bleed.

## Review of published data

Search of the literature revealed 1 post-hoc analysis of HAVEN 1–4 studies (23), 1 phase IV study which enrolled 11 children (14 total patients) (24), and 13 pediatric case series or case reports.

### Summary of HAVEN studies 1–4

During the 4 HAVEN trials, 126 patients underwent 233 surgical procedures including 215 minor procedures (115 patients, 64 patients with inhibitors) and 18 major surgeries (18 patients, 10 patients with inhibitors). Overall, 141/215 minor procedures (65.6%) received no pre-operative FVIII or rFVIIa and 128 of these procedures (85.8%) did not require post-operative FVIII or rFVIIa. In the major surgeries, 15 patients (83.3%) received additional FVIII or rFVIIa treatment and 12 patients had no postoperative bleeding. Having an inhibitor vs. no inhibitor had no effect on rate of postoperative bleeding, nor did receipt of pre-operative FVIII or rFVIIa (23).

The two most common minor procedures were dental (62 procedures; 29 extractions, 15 endodontic procedures, 7 dental implants) and central line procedures (29 removals, 7 placements). Of the 40 procedures performed without prophylactic FVIII or rFVIIa, 13 (32.5%) were associated with post-operative bleeds, 9 (27.5%) of which were treated and 4 of which did not require factor concentrate. Prophylactic FVIII or rFVIIa was administered in 22 dental procedures; 11 (50%) of these procedures had associated post-operative bleeds, 5 (22.7%) of which were treated and 6 were untreated bleeds. Antifibrinolytic agents were given in 32 procedures either as an adjunctive or as the singular hemostatic agent. Seventeen patients received antifibrinolytic therapy as the sole hemostatic agent; 10 of these procedures were not associated with a bleed.

Of the 36 central line procedures, 35 occurred in FVIII inhibitor patients. The one patient without inhibitors did not receive prophylactic factor nor did they have postoperative bleeding. Nine procedures were managed with prophylactic FVIII or rFVIIa, 2 of which required further treatment for bleeding. The 27 procedures that did not receive prophylactic FVIII or rFVIIa had 1 treated bleed and 1 untreated bleed. Antifiribrinolytic agents were given as the only treatment in 12 central line procedures and 8/12 had no bleeding complications.

While specific pediatric data was not separately reported, data from the 88 FVIII inhibitor patients enrolled in HAVEN 2 showed that 43 had a central line and 21 underwent central line removal. In this cohort, 17 had no prophylactic rFVIIa, and 1 patient had a treated postoperative bleed. Four patients received preoperative rFVIIa and none had postoperative bleeding. Specifics on other pediatric procedures were not reported (18).

Overall, minor surgery was well-tolerated with low rates of bleeding regardless of whether prophylactic infusions were given. A similar pattern was seen in the pediatric patients in these studies although analysis was limited and not specific for this age group.

### Phase IV study

A phase IV, multicentre, open-label study of emicizumab prophylaxis in persons with hemophilia A with or without FVIII inhibitors undergoing minor surgery was conducted from June 2018 to March 2020. The mean age of patients who participated in the trial was 11 (range: 5–22). Patients receiving emicizumab who were scheduled to undergo minor surgery within 60 days of enrolment and were planned to receive emicizumab for ≳1 month following surgery were eligible to participate in this study. The primary objective was to compare the percentage of participants who required FVIII or rFVIIa for surgery-related bleeding until discharge from surgery to those who did not require FVIII or rFVIIa, and to report the occurrence of bleeding and FVIII or rFVIIa use after discharge. Perioperative administration of FVIII, rFVIIa and antifibrinolytics was at the discretion of the treating physician (24).

Overall, 14 patients enrolled in this study with 13 undergoing minor surgical procedures (11 were children). Minor procedures performed in this study included CVAD removal (*n* = 11) and simple dental extractions (*n* = 2). Only 3/14 participants did not have FVIII inhibitors. rFVIIa was administered intra-operatively in three minor surgeries, although only one was for treatment of an intraoperative bleed. Post-operative bleeding occurred in three participants, two of whom received rFVIIa intraoperatively; all three patients received post-operative rFVIIa. The study was terminated early due to low enrollment and limited variety of surgical procedures.

Of the 11 patients who underwent central line removal, bleeding occurred in 2; 1 postoperative bleed and 1 intra-operative bleed. Both received intra and post-operative rFVIIa. One additional patient undergoing central line removal received rFVIIa immediately prior to surgery (and was classified as receiving intra-operative factor) and had no postoperative bleeding. The 8 other central removals did not receive preoperative or intra-operative FVIII or rFVIIa and had no postoperative bleeding. Both dental extractions had postoperative bleeding although only 1 was a treated bleed.

Early termination due to few patients undergoing minor surgeries and low enrollment numbers limited the generalizability of this study. Conclusions were difficult to draw in this small study although the results were similar to those reported in the HAVEN studies and other cohort studies. Most pediatric patients in this study tolerated central line removals without the need for preoperative or intraoperative FVIII or rFVIIa replacement. The authors still advised clinical judgment as the major deciding factor for when to give preoperative or intraoperative factor and to continue to coordinate surgical care with expert clinicians in hemophilia care.

### Case series and reports

Thirteen publications reported surgical approaches and outcomes in CwHA. Similar to previous publications, the most common minor surgical procedure was central line removal or insertion (82 patients, 79 removals, 3 insertions, Table 1). Post-operative bleeding was minimal and occurred at the same rate regardless of administration of prophylactic FVIII or rFVIIa or no preoperative factor. The most common bleeding complications were hematoma development and bleeding from the surgical site (25–39).

**Table 1:** Reports of Central Venous Access Device (CVAD) surgeries in CwHA on emicizumab.

No preoperative factor				Received preoperative factor			
Study	N^*^	Postoperative outcome	Post-operative Therapy	Study	N^*^	Postoperative outcome	Postoperative Therapy
McCary et al. (26)	4	No bleeds		McCary et al. (26)	17	3 hematoma	1 received rFVIIa
	3 non-inhibitor				12 non-inhibitor	(1 inhibitor, 2 non-inhibitor)	2 received rFVIII
	1 inhibitor				5 inhibitor		
Hassan et al. (27)	10	No bleeds	All received tranexamic acid × 7 days	Hassan et al. (27)	1 inhibitor	No bleed	Tranexamic acid pre and post-operative
	7 non-inhibitor						
	3 inhibitor						
Lewandowska et al. (25)	8	3 bleeds	Received rFVIII	Lewandowska et al. (25)	1 inhibitor	1 bleed	Received rFVIIa
	5 non-inhibitor						
	3 inhibitor						
Zimowski et al. (35)	1 inhibitor	No bleed		Zimowski et al.(35)	1 inhibitor	1 hematoma	Received rFVIIa
Badle et al. (38)	10 inhibitor	1 untreated bleed	All received tranexamic acid	Cohen et al.(29)	5 non-inhibitor	1 hematoma	All received planned rFVIII
Swan et al. (28)	10	1 bleed	Received only tranexamic acid	^@^Batsuli et al.(33)	5 inhibitor	No bleeds	All received planned rFVIIa (1) or rFVIII (4)
	8 non-inhibitor						
	2 inhibitor						
Barg et al. (30, 31)	2 non-inhibitor	No bleeds		Barg et al.(30)	5	No bleeds	
					3 non-inhibitor		
					2 inhibitor		
				Lockhart et al.(36)	2 inhibitor	No bleeds	
Total	45	4 TB		Total	37	1 TB	
		1 UTB				5 treated Hematoma	

^*^ inhibitor patients include current and historic inhibitors.

^@^ 3 patients had central line insertion, 2 had central line removal.

rFVIIa, recombinant factor VIIa; rFVIII, recombinant factor VIII; TB, treated bleed; UTB, untreated bleed; N, number of patients.

Twenty-one additional minor surgical procedures were reported and are described in Table 2. The majority of these procedures (15/21) were performed with pre-operative FVIII or rFVIIa administration and resulted in minimal bleeding. Four of these procedures also received planned FVIII or rFVIIa post-operatively and reported no bleeding.

**Table 2:** Reports of other minor surgical procedures in CwHA on emicizumab.

	N	Preoperative therapy	Postoperative outcome	Postoperative therapy
***Dental Procedures***				
McCary et al. (26)	2 inhibitor	1 pdVWF + ACA	No bleeds	None
		1 ACA		
Lewandowska et al. (25)	1 inhibitor	rFVIIa	Bleeding	rFVIIa × 3 doses, ACA × 10 days
	1 non-inhibitor	rFVIII	No bleed	ACA × 2 days
Barg et al. (30)	1 non-inhibitor	None	None	None
Hassan et al. (27)	1 non-inhibitor	rFVIII	No bleed	TXA × 5 days
***Circumcision***				
Barg et al. (30, 31)	1 inhibitor	None	Severe bleeding	rFVIIa
				Red blood cell transfusion
Batsuli et al. (33)	1 inhibitor	rFVIII	No bleed	rFVIII × 1 dose (planned)
Kavakli et al. (39)	1 inhibitor	none	No bleed	Fibrin glue during operation
				Tranexamic acid × 7 days
Zulfiker et al. (40)	1 inhibitor	Tranexamic acid	No bleed	Tranexamic acid × 10 days
***Minor Orthopedic Procedures***				
Lewandowska et al. (25)	1 non-inhibitor	rFVIII	No bleed	rFVIII × 1 dose
*Right ankle foreign body removal*				
McCary et al. (26)	1 non-inhibitor	rFVIII	No bleed	None
*Right elbow arthroscopy with limited synovectomy*				
***Other minor surgical procedures***				
Barg et al. (30)				
* Sutures for head trauma*	1 inhibitor^*^	FVIII^#^	No bleed	None
* Facial laceration suture*	1 inhibitor^*^	FVIII^#^	No bleed	None
* Debridement of right hand*	1 inhibitor^*^	FVIII^#^ + rFVIIa	Bleeding	None
* Gastroscopy and colonoscopy*	1 inhibitor	FVIIa	No bleed	None
McCary et al. (26)				
* Bilateral ear tube removal*	1 non-inhibitor	rFVIII	No bleed	None
* Bilateral laparoscopic inguinal hernia repair*	1 non-inhibitor	pdvWF	No bleed	None
* PICC line placement*	1 non-inhibitor	pdvWF	No bleed	None
Lewandowska et al. (25)				
* Cardiac catheterization, removal of central line fragment*	1 non-inhibitor	rFVIII	No bleed	None
Cohen et al. (29)				Planned rFVIII dosing: rFVIII Q8H × 2 doses rFVIII Q12H × 2 doses rFVIII daily × 3 days
* AV fistula ligation*	1 non-inhibitor	rFVIII	No bleeding	

rFVIIa, recombinant factor VIIa; rFVIII, recombinant factor VIII; N, number of patients; ACA, amino-caproic acid; pdvWF, plasma-derived von Willebrand factor concentrate; PICC, peripherally-inserted central catheter; TXA, tranexamic acid.

*low-titer inhibitor (<5 Bethesda Units).

^#^ type of FVIII (recombinant or plasma-derived not specified).

Four circumcision procedures were reported and all 4 had different perioperative treatment regimens including prolonged antifibrinolytic treatment with or without fibrin glue (2 patients, no postoperative bleeding) (32), rFVIII preoperative with 1 planned dose postoperative (1 patient, no postoperative bleeding) (33), and no preoperative treatment (1 patient, major postoperative bleeding requiring rFVIIa and packed red blood cell transfusion) (31).

Five publications reported 8 major procedures (4 orthopedic procedures, 2 ventriculo-peritoneal shunt revisions, 1 cleft palate repair) and are described in Table 3. All patients who underwent major surgery received prophylactic and prolonged postoperative FVIII or rFVIIa and all reported good outcomes with no intraoperative or postoperative bleeding (25–27, 29, 30, 34).

**Table 3:** Reports of major surgical procedures in CwHA on emicizumab.

	N	Preoperative therapy	Postoperative outcome	Postoperative therapy
Lewandowska et al.(25)				
* Patellofemoral ligament reconstruction*	1 non-inhibitor	rFVIII	No bleed	rFVIII daily×5 days
* Open reduction internal fixation of 5th phalangeal joint*	1 non-inhibitor	rFVIII	No bleed	rFVIII daily×1 day (q12 h × 2 doses)
McCary et al.(26)				For both:
* Spinal fusion*	1 non-inhibitor	rFVIII	No bleed	rFVIII daily to keep levels >50% × 7 days
* Ventriculoperitoneal shunt revision*	1 non-inhibitor	rFVIII	No bleed	
Barg et al.(30)				
* Ventriculoperitoneal shunt revision*	1 non-inhibitor	rFVIII	No bleed	Daily rFVIII × 4 days
Hassan et al.(27)		rFVII		rFVIII daily × 4 days
* Cleft palate repair*	1 non-inhibitor	Tranexamic acid	No bleed	Tranexamic acid × 7 days
Cohen et al.(29)				rFVIII Q12H × 3 days
* Complex orthopedic surgery*	1 non-inhibitor	rFVIII	No bleed	rFVIII daily × 3 days
Lefevre et al.(34)				rFVIIIFc by continuous infusion during surgery then daily × 6 days
* Left femoral osteotomy*	1 inhibitor	rFVIIIFc	No bleed	
* *				rFVIIa Q2H×24 h on day 7 based on inhibitor result and thrombin generation monitoring

rFVIIa, recombinant factor VIIa; rFVIII, recombinant factor VIII; rFVIIIFc, recombinant factor VIII with Fc fusion; N, number of patients.

## Discussion

Emicizumab, a bispecific monoclonal antibody which mimics the action of factor VIII has improved bleeding outcomes for patients with severe Hemophilia A. Its evolution has impacted the way providers manage patients with and without inhibitors; many have shifted away from routine use of factor VIII bypassing agents for patients with inhibitors. Data regarding surgical management and outcomes continues to evolve. The incorporation of emicizumab as prophylaxis for CwHA with or without inhibitors has represented a major shift in therapy. The experience around surgery for these children continues to evolve.

This review captured 103 minor surgeries that were reported in children on emicizumab prophylaxis. Nearly half (46.6%) of the procedures did not utilize pre-operative factor. Of the 48 surgeries that did not utilize additional hemostatic factor, 6 (12.5%) were associated with post-operative bleeds, one of which was major, requiring a red blood cell transfusion. Of the remaining 55 surgeries that utilized pre-operative factor prophylaxis, 6 (10.9%) also had post-operative bleeding.

The data demonstrates that many minor surgeries in children may be safely done with no additional factor prophylaxis or with one pre-operative factor dose. These included common minor surgeries such as dental extractions and central line removal, although the latter will likely decrease in incidence as more CwHA switch to emicizumab and no longer require regular venous access for factor prophylaxis or, in the case of inhibitor development, immune tolerance induction. Bleeding appeared to be similar between pre-treated and untreated patients. Management of post-operative bleeds varied between studies and included observation only and treatment with 1 dose of additional factor with or without adjunctive therapies such as antifibrinolytics.

Potential limitations of this review include possible publication bias such as case reports of surgery with bleeding complications being less likely to be published, and patients with a high clinical bleeding tendency not being included in surgery studies. We think these limitations are likely of low probability given that emicizumab was first used in patients with Hemophilia A with inhibitors which tend to have the highest bleeding tendency and these higher risk patients comprise the majority of the HAVEN study patients (23) and those reported in the case series (25–36).

While conclusions are limited based on available data, care needs to be taken in infants potentially owing to developmental differences in hemostasis (31), and possibly different metabolism or clearance of emicizumab. Further data should be forthcoming once the results of the ongoing HAVEN 7 study are reported (37). In this age group, until more information is known, provision of prophylactic factor and probably post-operative factor should be administered in situations where surgery is required.

For older children, what is the right approach? Should prophylactic factor be given prior to minor surgical procedures? Is it safe to not give prophylactic factor and instead observe postoperatively and give factor only if significant bleeding? Both approaches seem to be safe and without significant adverse events. These decisions require expertise from the hemophilia treatment team and close collaboration with the surgical team to create an optimal plan for each patient. The individual bleeding risk of the patient based on past bleeding pattern, FVIII inhibitor status, other medical conditions that may delay wound healing, and concomitant medical diagnoses also need to be considered when assessing risk of operative bleeding (25).

Giving prophylactic factor preoperatively could result in less factor required postoperatively, and emicizumab may provide enough FVIII equivalent levels to allow for adequate wound healing without re-bleeding. However, it will still be challenging to come up with strict guidelines as each minor surgery type may have different bleeding risks and many minor surgical procedures have minimal reported outcome data based on current publications.

The picture is clearer in major surgery and a similar approach as is done in patients with mild hemophilia should be the norm, namely a low threshold for additional factor replacement to ensure FVIII levels close to 80%–100% especially during surgery and in the immediate postoperative period. One possible advantage of emicizumab that we have observed in these situations is that additional factor administration post-operatively can likely be stopped earlier than in the past given the FVIII-equivalent levels emicizumab provides.

Identifying an appropriate perioperative hemostatic agent is important for optimizing hemostasis in patients on emicizumab undergoing major surgery especially as patients with active inhibitors should only receive rFVIIa due to the observed thrombotic complications in patients who received aPCC (17). This is a situation where laboratory monitoring (when available) to determine the presence or absence of an inhibitor in patients with a past history of inhibitors could be useful to determine appropriate factor replacement choice. Additional high-level laboratory monitoring such as thrombin generation assays may also be considered to assess bleeding and thrombosis risk, however this assay is often restricted to highly specialized centers and not widely available. Using such strategies could be considered especially for highly complex major surgeries and would require discussion, collaboration and forward planning with the Hemophilia, Laboratory Medicine, and Surgical teams. If patients have not been exposed to FVIII in a long time before surgery and they received FVIII replacement during and after surgery, testing for emergence of a new or previous inhibitor should be done to guide future factor therapy.

With the widespread adoption of emicizumab worldwide, a new cohort of CwHA who have had none or minimal factor exposure is expected to grow. This “factor naive” group will present different challenges when faced with surgery, especially when deciding on supplemental FVIII. Should the approach to surgery be similar to the decisions around factor administration in children with mild hemophilia A? One difference is that unlike mild HA patients who can sometimes use desmopressin as a way to increase their baseline FVIII level, in severe HA patients on emicizumab, additional factor administration before and possible following surgery will need to be considered if higher FVIII levels are needed for the surgical procedure.

One other major shift expected with widespread adoption of emicizumab is reduction in central line procedures as fewer patients opt for regular FVIII replacement as their prophylaxis regimen and fewer patients opt for immune tolerance induction therapy for inhibitor eradication. This likely means that there will be fewer pediatric surgery procedures overall but will also mean a shift in the type of surgical procedures seen in CwHA to more what is commonly seen in the pediatric age groups namely trauma-related surgeries, emergency procedures, otolaryngology procedures such as tonsillectomy, adenoidectomy and myringotomy tube placement, and repair of congenital malformations. More data and experience with these types of procedures will need to be collected to understand the best approaches to safely performing these surgeries with minimal to no bleeding. Likely, many of these types of surgeries would be classified as major surgery and hence require additional FVIII coverage.

Development of specific surgical guidelines is difficult given the variety of surgical procedures and their different bleeding risks. However, the following approach is suggested:

1. Close collaboration between the surgical team and hemophilia clinicians involved in the care of the child with hemophilia A.
2. Identify the bleeding risk for the specific procedure.
3. Consider if the FVIII equivalent levels provided by emicizumab are sufficient for this procedure to occur without bleeding or if additional FVIII is required to increase levels desired.
4. Consider if a dose of FVIII or rFVIIa preoperatively would greatly reduce the chance of needing multiple doses of replacement postoperatively.
5. For CwHA with inhibitors, consider rFVIIa only [given the reported serious adverse events with aPCC and emicizumab (17)] unless no inhibitor is detected prior to surgery. In this case, dosing with FVIII could be done but only under supervision of the Hemophilia care team.
6. Consider specialized laboratory testing to help guide therapy in complex major surgeries. Coordination and forward planning with surgical teams, laboratory, and the Hemophilia care team is essential in this case.
7. Be prepared and have a plan in case of bleeding.
8. Prepare parents for the possibility of a bleeding emergency after discharge with clear plans on when to present for re-evaluation.

## Conclusion

The use of emicizumab as prophylaxis for CwHA has revolutionized hemophilia A care. Understanding the safest approaches to major and minor surgeries in children is important to minimize bleeding complications during and after surgery. While no specific guidelines exist for how to best manage surgeries in the pediatric population, recent publications have shown that most minor procedures can be safely completed without factor prophylaxis pre-procedure. While some procedures may necessitate the use of prophylactic factor pre-operatively, both approaches seem to result in mild or no postoperative bleeding for almost all minor procedures. Major surgeries still require ongoing factor administration postoperatively and duration of postoperative factor should continue to be guided by desired FVIII levels based on days since surgery occurred. One benefit of emicizumab is that less postoperative factor may be needed given the FVIII equivalent levels reached with emicizumab. Ongoing collaboration between the surgical teams, laboratory, and Hemophilia care team remains crucial. Further understanding of surgical outcomes for CwHA on emicizumab in trauma surgery, repair of congenital malformations and other common pediatric surgeries will need to continue. Collection of these outcomes through pre-planned national or international registries would be the best way to further this understanding.
